# Supplementary material for: Gender Differential Morbidity in Quality of Life and Coping Among People Diagnosed with Depression and Anxiety Disorders
Source: Healthcare (Basel). 2025 Mar 23;13(7):706. doi: 10.3390/healthcare13070706 (PMC11988614; doi:10.3390/healthcare13070706)
Supplement: Supplementary file 1 [file healthcare-13-00706-s001.zip › healthcare-3524725-supplementary.pdf]

Table S1: Description of the variables (if required) of the instruments used in the study

	**Sociodemographic Questionnaire**	
**Uses of time**	Household chores	Cleaning, tidying up, doing physical labour and housekeeping.
	Leisure activities	Meeting friends or relatives, hobbies, entertainment…
	Personal self-care	Beauty self-treatment, hair and skin caring, makeup… (Hair salon, spa, facial treatments or similar activities are not included).
	Care of dependents	Assisting with dressing, undressing, feeding. Helping with hygiene, assisting with transfers…
**Weekly Physical activity** [31]	None	Null activity.
	Low	Low activity, sedentary lifestyle.
	Moderate	Daily walking / Doing moderate exercise 3 or more days for at least 20-30 minutes per day.
	High	Doing vigorous intensity activities at least 3 days / every day of medium intensity exercise.
	**LSB-50** [32]	
**Psychoreactivity**	Sensitivity in self-perception in relation to others and one's own image.	
**Hypersensitivity**	Interpersonal and intrapersonal sensitivity regarding fixation or focusing.	
**Obsession-compulsion**	Presence of obsessions and continuous doubts that remain in thought, as well as rituals or compulsions.	
**Anxiety**	Manifestations of generalized anxiety disorder, panic disorder, phobic anxiety and symptoms of fear or irrational fear.	
**Hostility**	Presence of reactions of loss of emotional control with sudden or continuous manifestations of aggressiveness, anger, rage or resentment.	
**Somatization**	Somatic or bodily discomfort due to psychosomatic processes or medical pathology. It includes imbalances of the autonomic nervous system affecting the respiratory, muscular, cardiovascular and gastrointestinal systems.	
**Depression**	Presence of characteristic symptoms such as sadness, hopelessness, anhedonia, anergy, impotence, or self-destructive ideation, including guilt.	
**Sleep disturbances**	Specific alterations relevant from the point of view of health and well-being.	
**Extended sleep disturbances**	Presence of sleep disturbances with manifestations of the Anxiety and Depression scales that are clinically associated with sleep. This scale allows to distinguish between primary and secondary sleep disturbances.	
**Psychopathological Risk Index (IRPSI, by its Spanish acronym)**	In the psychopathological clinical population, it evaluates the presence of symptoms associated with devaluation, misunderstanding, fear, somatization, hostility, and suicidal ideation.	
**Global Severity Index (GLOBAL)**	Degree of global psychopathological affectation. Global measure of the intensity of mental and psychosomatic suffering.	
**Number of suffered symptoms (NUM)**	Amplitude and diversity of the symptomatology of the subject.	
**Intensity Index of suffered symptoms (INT)**	Specific symptomatic intensity, not of the set of evaluated symptoms (it is reflected in the GLOBAL index).	
	**EQ-5D-5L** [34]	
**Mobility**	Walking ability and motor independence.	
**Self-care**	Hygiene, washing and dressing oneself.	
**Daily activities**	Working, studying, household chores, doing family activities or other activities in leisure time.	
**Pain or discomfort**	Associated symptomatology.	
**Anxiety or depression**	Associated symptomatology.	

COPE-28 [38]		
**Cognitive coping**	**Active coping**	Initiating direct actions, increasing own efforts, eliminating or reducing the stressor.
	**Planning**	Thinking about how to deal with the stressor. Planning action strategies, the steps to take and the direction of the efforts to be made.
	**Positive reframing**	Looking for the positive and favorable side of the problem and trying to improve or grow from the situation.
	**Humour***	Making jokes about the stressor or laughing at stressful situations, making fun of it.
	**Behavioural disengagement**	Reducing efforts to deal with the stressor, including giving up effort to achieve the goals with which the stressor interferes.
	**Acceptance**	Understanding the fact of what is happening, that it is real.
**Coping through social support**	**Instrumental support**	Seeking help, advice, information from people who are competent about what should be done.
	**Emotional support**	Getting emotional support of sympathy and understanding.
	**Venting**	Increased awareness of one's own emotional distress, accompanied by a tendency to express or discharge those feelings.
**Blocking coping (avoidance)***	**Self-distraction**	Focusing on other projects, trying to distract yourself with other activities, to try not to focus on the stressor.
	**Denial**	Denying the reality of the stressful event.
	**Self-blame**	Criticizing and blaming yourself for what happened.
	**Use of substances**	Taking alcohol or other substances to feel good or to help cope with the stressor.
**Spiritual coping**	**Religion**	Tendency to turn to religion in times of stress or increasing participation in religious activities.

* Humor subscale is also present at the Avoidant coping group.
